# Supplementary material for: Increased CD4+CD8+ Double Positive T Cells during Hantaan Virus Infection
Source: Viruses. 2022 Oct 13;14(10):2243. doi: 10.3390/v14102243 (PMC9611689; doi:10.3390/v14102243)
Supplement: Supplementary file 1 [file viruses-14-02243-s001.zip › viruses-1909936-supplementary.pdf]

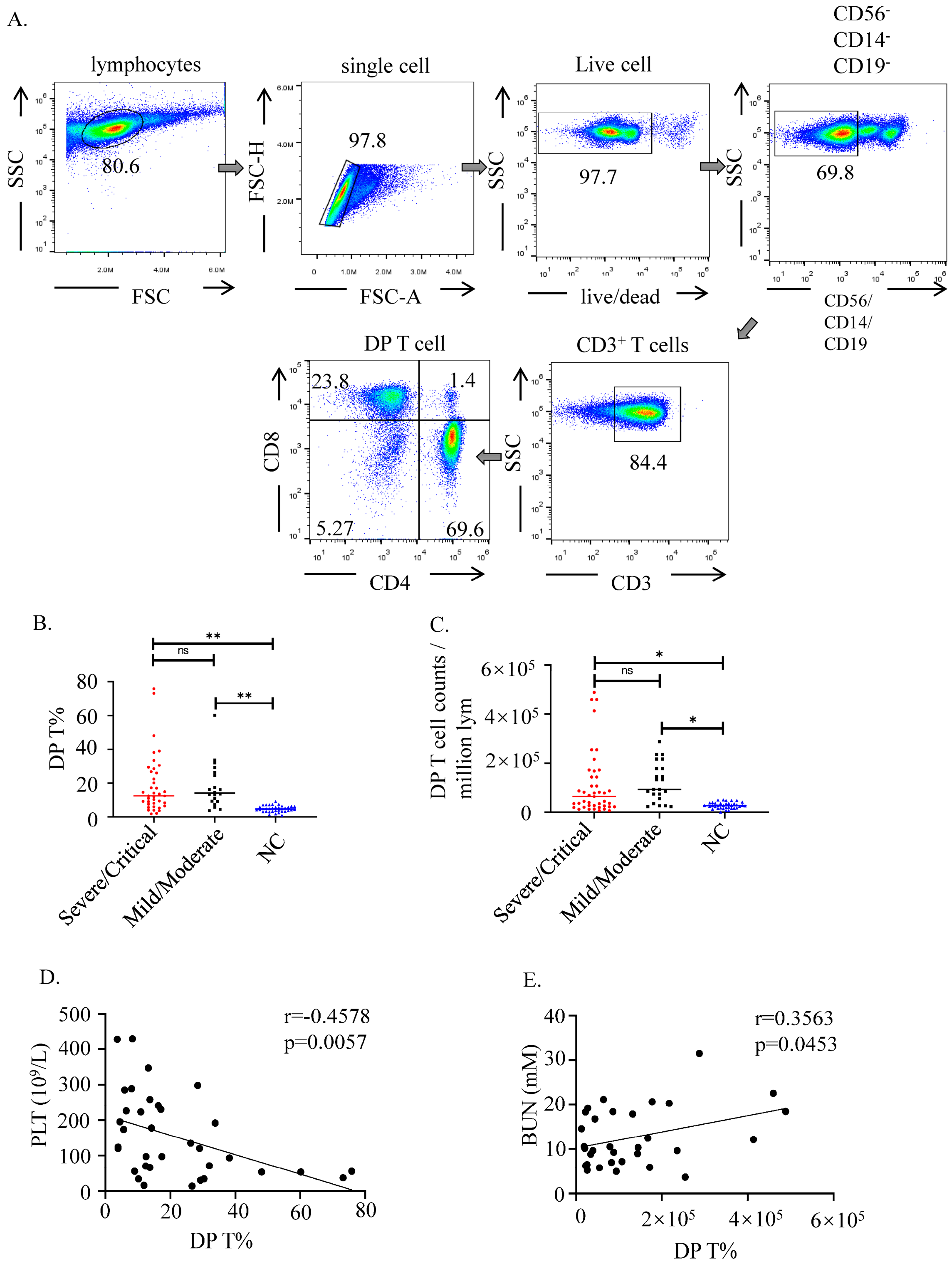

Figure S1. The DP T cells in HFRS patients under different severity. A. Flow cytometric plots showed the gating strategy of DP T cells. Statistical analysis showed the comparison of percentage of DP T cells (B) and cell counts of DP T cells (C) in HFRS patients with different severity and NC. (For HFRS, N=59; For NC, N=36) D. The correlation between the percentage of DP T cells and platelet counts (PLT). E. The correlation between the DP T cell counts and blood urea nitrogen (BUN) in HFRS patients (N=32). *p<0.05; **p<0.01; "ns" means no significance. Pearson correlation coefficient r values and respective p values are shown on each figure.

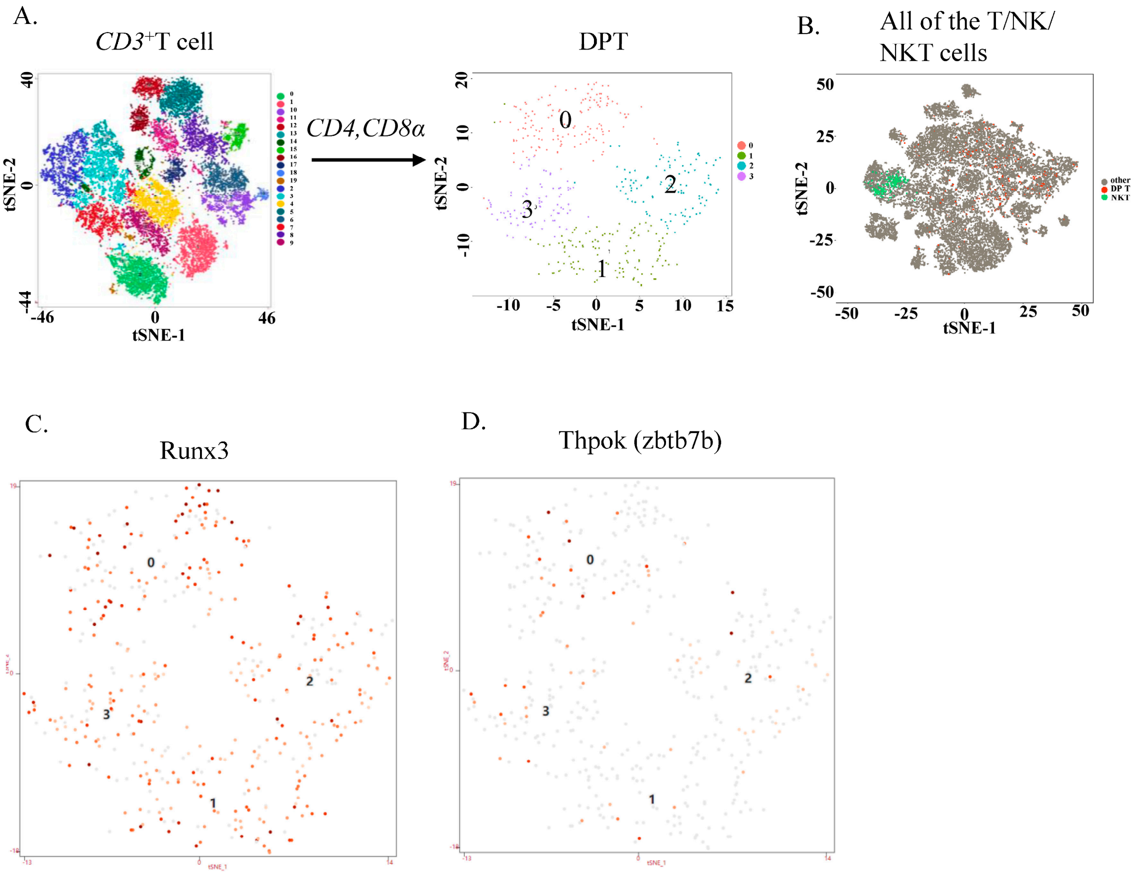

Figure S2. The scRNA-seq data showed the gene expression of DP T cells. A. scRNA-seq screened out DP T from T cells with expression of CD4 and CD8 simultaneously. B. The distribution of DP T cells and NK T cells in all the T/NK/NKT cells' clusters. The expression of Runx3 gene (C) and Thpok gene (D) in DP T cells. Numbers in dot plots denote cluster.

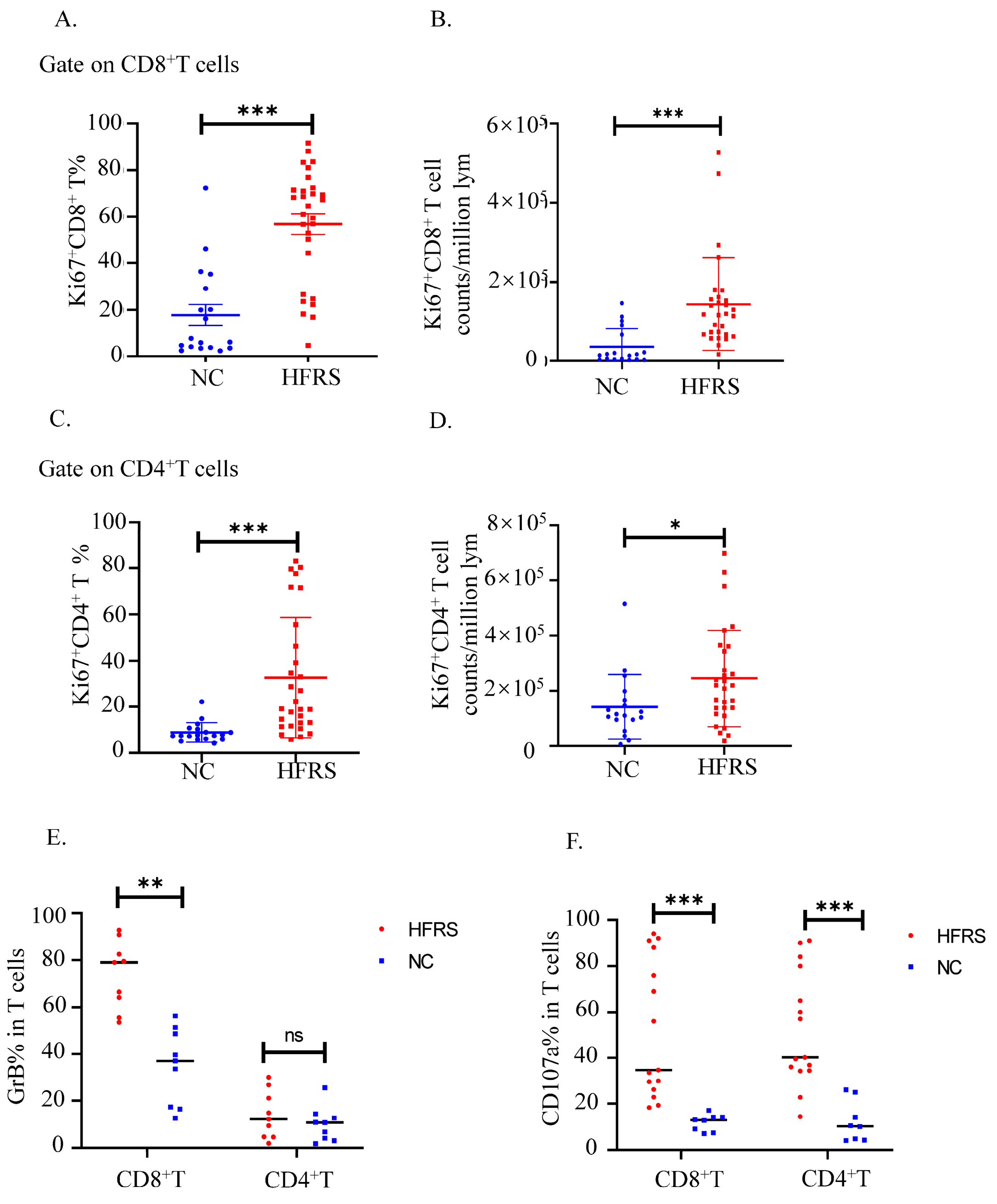

Figure S3. The proliferation and cytokines' production of conventional single positive T cells. A-B. The comparison of percentage (A) and cell count (B) of Ki67$^+$CD8$^+$T cells between HFRS patients and normal controls (NC). C-D. The comparison of percentage (C) and cell counts (D) of Ki67$^+$CD4$^+$T cells between HFRS patients and NC. (For HFRS, N=29; For NC, N=18). E-F. The comparison of the production of Granzyme B (GrB) (E) (N=9) and CD107a (F) (For HFRS, N=15; For NC, N=8) in CD8$^+$T cells and CD4$^+$T cells between HFRS patients and NC. "ns" means no significance. *$p < 0.05$, **$p < 0.01$, and ***$p < 0.001$.

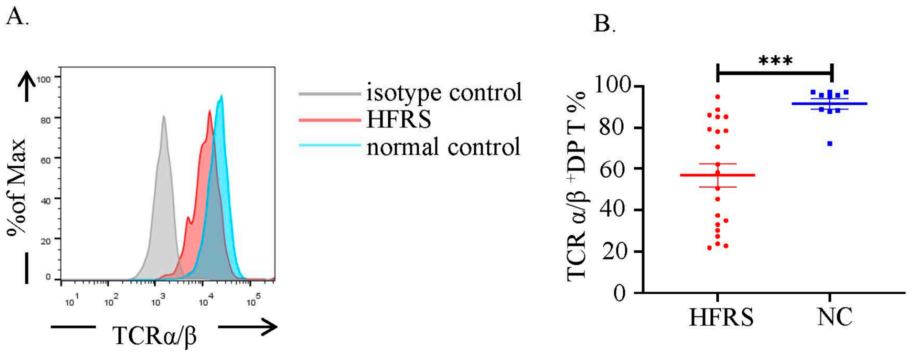

Figure S4. The expression of TCRα/β chain in DP T cells. A. The representative histogram showed the expression of TCRα/β chain in DP T cells from both HFRS patients and normal controls (NC). B. The statistical analysis of the TCRα/β$^+$DP T cells% in both HFRS patients and NC (For HFRS, N=21; For NC, N=10). ***p< 0.001.

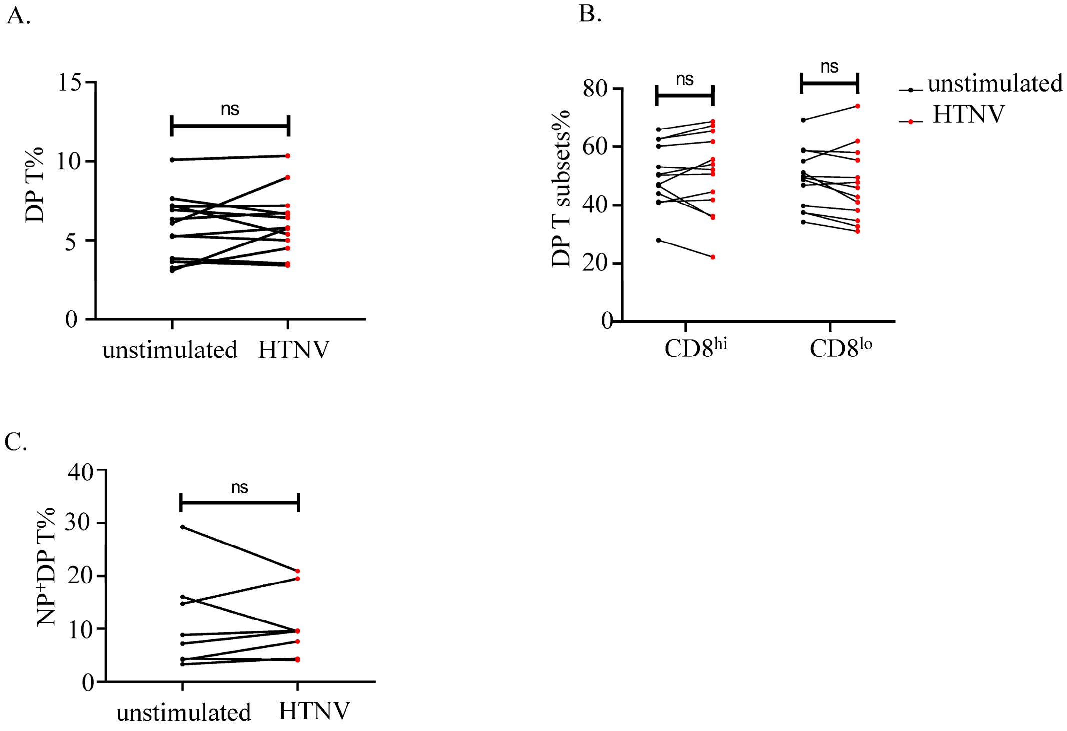

Figure S5. The change of DP T cells during *in vitro* infection. A. The statistical analysis showed the comparison of the percentage of DP T cells in unstimulated group and HTNV infection group from *in vitro* infection assay (N=13). B. The comparison of CD8^{hi} and CD8^{lo} subsets in unstimulated group and HTNV infection group from *in vitro* infection assay (N=13). C. The protein levels of nucleocapsid protein (NP) of HTNV with or without *in vitro* HTNV infection (N=8). "ns" means no significance.

Supplementary Table S1: Information of antibodies used in the study.

antibody	fluorescent	Clone No.	company
CD3	PE-Cy7/percp-cy5.5	SK7/OKT3	Biolegend
CD4	PerCp-Cy5.5	RPA-T4	Biolegend
CD8	APC-Cy7/APC/BV510	SK1/RPA-T8	Biolegend
Ki67	Alexa 488	D3B5	Cell Signaling Technology
CCR7	FITC/BV421	G043H7	Biolegend
CD45RA	APC	HI100	Biolegend
Granzyme B	FITC	GB11	Biolegend
CD107a	PE	H4A3	
IFN-γ	APC	4S.B3	Biolegend
TCRα/β	FITC	IP26	Biolegend
CD56	FITC	HCD56	Biolegend
CD14	FITC	M5E2	Biolegend
CD19	FITC	HIB19	Biolegend
HTNV-NP	FITC	1A8	Provided by the Department of Microbiology of FMMU
